# The Use of Multicriteria Inference Method to Identify and Classify Selected Combustion Engine Malfunctions Based on Vehicle Structure Vibrations

**DOI:** 10.3390/s21072470

**Published:** 2021-04-02

**Authors:** Krzysztof Prażnowski, Andrzej Bieniek, Jarosław Mamala, Adam Deptuła

**Affiliations:** 1 Faculty of Mechanical Engineering, Opole University of Technology, 45-758 Opole, Poland; a.bieniek@po.edu.pl (A.B.); j.mamala@po.edu.pl (J.M.); 2 Faculty of Production Engineering and Logistic, Opole University of Technology, 45-758 Opole, Poland; a.deptula@po.edu.pl

**Keywords:** internal combustion engine, logic tree, fault algorithm

## Abstract

Internal combustion engines are among the most commonly used propulsion units for drive systems in various industries such as land transportation, maritime transportation, and power generation. Their operation involves a continuous change of technical condition as a result of not only the combustion process but also their operation under conditions of variable load or ambient impact. It is therefore important to monitor the technical condition of internal combustion engines to ensure high performance and reliability over their lifetime. The article presents the test results obtained from incorrect operation of an internal combustion engine as a result of forced failures of the ignition and injection system. On this basis, a multicriteria comparison of the signal analysis of engine block vibrations was made, after the transformation of the signal from the time domain to the frequency domain, by using the induction technique obtained from the operation of decision tree algorithms. For this purpose, the amplitude spectrum in the frequency domain, scaled to absolute values of discretization for which teaching and testing data tables were created for successive harmonics, was determined for the engine block vibration signal being tested. On the basis of the developed algorithm using decision trees, a multicriteria data table was analyzed for which a compatibility path for the analyzed engine block vibration signal is created. In this way, it is confirmed with a specified degree of effectiveness, depending on the point of operation of the engine resulting from its crankshaft speed, that there is a possibility of detecting a preset ignition or injection system malfunction in the technical condition of the internal combustion engine with a probability up to about 72%.

## 1. Introduction

The internal combustion engine is a strongly nonlinear object, consisting of many subcomponents, which, combined together, are responsible for its proper functioning. It is their interaction that results in obtaining parameters of engine operation; however, its operation is linked with body vibrations which are minimized in the design process. The engine is a real object, in which random irregularities can occur, resulting from the poor quality of fuel or from control system errors when they have a repetitive character. Such faults cause the divergence of the indicators of engine operation from the expected values, causing a decrease in power, an increase in fuel consumption, an increase in exhaust emissions, and changes in the noise spectrum but also in the occurrence of intense vibrations on the engine body. A frequently signaled malfunction related to the operation of the engine is the occurrence of knocking combustion and the so-called misfire. The causes of the detected combustion errors are not unambiguously determined and may be related, depending on the type of internal combustion engine, to the lack of fuel injection, inappropriate fuel atomization, bad air-fuel ratio (AFR), or lack of ignition spark and other errors, indicating its poor technical condition. From the point of view of the durability and reliability, as well as for environmental reasons, it is important to detect the errors as early as possible. For this reason, many research centers conduct research work related to the diagnosis of this type of defects in a reciprocating internal combustion engine, where the vibrations of individual engine components or their fitting are analyzed.

Barelli et al. [1] analyzed the quality of the combustion process and developed an innovative diagnostic system using DWT (discrete wavelet transform) analysis for cogeneration reciprocating engines. This system is based on the evaluation of the energy content of the vibration signal directly acquired on the cylinder heads through the discrete wavelet transform technique and the Parseval’s theorem. Grajales et al. [2] dealt with the diagnosis of internal combustion engine faults caused by the misfire, where they based the diagnosis on the analysis of the engine vibration signals using envelope, peak energy, and peak longitudinal acceleration techniques. Jianfeng et al. [3] presented a method of diagnosing the misfire based on gradient boosting using a time and frequency analysis of vibration signals using a triaxial accelerometer. For an accurate and timely diagnosis of diesel engine misfire, this article developed a novel diagnostic method combining a few methods. Thus, multi-synchro-squeezing (MSST), local linear deposition (LLE), and extreme gradient gain (XGB) were combined. The results confirm a very high classification accuracy of the proposed algorithm based on gradient boosting up to 99.93%.

Taghizadeh-Alisaraeii Mahdavian’s work [4] is focused on the diagnosis of combustion engine injector fault and corresponding vibration amplitudes and frequencies likely to cause the knock phenomenon. Welch test, short-term Fourier transform (STFT), Wigner–Ville distribution (WVD), and Choi-Williams distribution (CWD) were employed for a detailed scrutiny of vibrations generated by the engine under load. Komorska [5] proposed to build a statistical model with abstractive parameters that could be, for instance, a periodically time-varying autoregressive model AR for the diagnosis of given damages, like valve clearance change or valve burning. Identifying model consists in finding periodically time-varying coefficients of the equivalent filter.

Applying the STFT method to the diagnosis of IC engine valve clearance was shown by Jedlinski et al., who analyzed the transformer window and the dominant frequencies. Due to the signal dependence, the Kalman filter was used to analyze the vibration signal for the diagnosis of IC engine valve clearance, which was presented by Puchalski [6]. The occurrence of knock combustion was determined by the new knock index by Bares et al. [7]. In this work, Bares at al. proposed analyses of the frequency spectrum of the pressure signal in two locations, i.e., near the maximum heat release and near the end of combustion, using the fast Fourier transform and a window function which is compared with the classical MAPO definition consisting in finding the maximum pressure oscillation in the time domain. Genga et al. [8] published an analytical model of nonstationary engine vibrations. Attention was paid to the time-varying vibrations with specific properties which are important for the transformation of the vibration signal from the time domain to the frequency domain, while maintaining its dominant properties. An autoregressive model based on pseudo-Wigner–Ville distribution (AR-PWVD) was designed and applied into this research work for detecting insufficient combustion and outlet valve leakage.

Regardless of the research possibilities to detect a defect, some researchers, such as Ahirrao et al. [8], pointed out to the effect of the engine fixture to the frame and the structural vibrations transmitted by the engine-frame assembly. Prażnowski at al. [9] indicate that it is possible to diagnose selected engine faults through vehicle structure vibration analysis using STFT spectrum and inference matrix.

Many researchers propose the use of advanced detection algorithms in the form of artificial intelligence, including neural networks, to diagnose engine failure in machines. Huang and Liu [10] proposed a multifeature fusion model based on Dempster–Shafer evidence theory combined with a particle swarm optimization algorithm and artificial neural network (PSO-ANN). The proposed model in comparison with the k-nearest-neighbors method may effectively improve the accuracy of damage forecasting. In turn, Gong et al. [11] proposed a novel method: that is, they improved the convolutional-neural-network–support-vector-machine (CNN–SVM) method. This method improves the traditional convolutional neural network (CNN) model structure by introducing the global average pooling technology and SVM. This paper proposes a novel method, namely an improved convolutional-neural-network–support-vector-machine (CNN–SVM) method. 

In order to analyze the identification of defects, other works propose to use the structure of decision trees for diagnostics of fault conditions and reliability. A linear model tree (LMT) was suggested by Sharma et al. [12] after comparing the competencies of various decision tree algorithms available. LMT algorithm offers high overall classification accuracy with the value of 100% in differentiating between normal and fault conditions. The use of vibration signals from the engine block secures a great accuracy and a lower cost. Wang at al. proposed a novel method named conditional inference tree to conduct the reliability analysis [13]. In [14,15], the theory of rough sets with high classification efficiency for analyzing the technical condition of bearings was presented. The decision to continue using the vehicle is based on the results of periodic inspections. Other papers present the use of classification methods for analyzing financial data [16,17].

In the induction and classification of knowledge acquisition, the decision consists of fragments describing the essence of the action to be taken. With the development of information technology, the nature and possibilities of optimization methods and decision support systems are changing. There is a wide range of research into the development of methodologies supporting decision-making processes and management control, design methodologies, and systems of various complexity, including artificial intelligence. A special direction of development that strengthens the role of classification systems is the combination of various methods of processing, inference, and seeking knowledge developed separately under artificial intelligence into one coherent hybrid consulting system. In particular, there are two general approaches to creating hybrid decision support systems: CI—computational intelligence and SC—soft computing [18]. In the case of CI, the criteria are selected by mathematical methods: neural networks, genetic algorithms, fuzzy logic, evolutionary, and heuristic programming. The second approach (SC) is to build hybrid systems for the use of artificial intelligence. It is assumed that existing advisory systems process additional structured information into additional structures, hierarchies, and semantic algorithms. When analyzing the state of research using decision support and decision making, one can observe the continuous development of computational intelligence methods, i.e., neural networks, fuzzy logic, rough sets, expert systems, and the main one of these methods, i.e., the hybrid method. This also applies to multicriteria decision-making methods developed, e.g., by Trzaskalik [19]. The work contains specific methods, including: AHP, ANP, ELECTRE, PROMETHEE, verbal methods, TOPSIS, BIPOLAR, and alternative methods used based on the risk analysis.

At the same time, the state of the art in machine learning is constantly increasing. It covers the problems of constructing systems whose operation increases with the experience represented by the set of teaching examples. In this area, particular attention should be paid to the further developments [20,21,22,23]. In the technical problems under consideration, one of the methods of classification of information and decision support is the method of inductive rule generation using decision trees. In induction, the entropy measure is used to determine the most significant attribute. Inductive decision trees can be compared in the process of classification, prediction, and determination of the importance of decision variables with multivalue logical trees [24]. In a broader sense, it can be claimed that inductive decision trees are a special case of modified tree structures [24,25].

There are many works presenting the use of inductive decision trees, multivalue logical trees (also with coefficients), and multivalue logical equations as decision support tools in the discrete optimization and determination of decision variables [26,27,28]. Classification methods found wide application in the analysis and diagnostics of internal combustion, electric, and hybrid engines. For example, Wu and Liu [29] proposed a system for diagnosing damage to internal combustion engines using wavelet packet transformation (WPT) and artificial neural network (ANN) techniques [30,31].

The paper [32] presents a method of diagnosing damage using error tree (binary) and Bayesian networks (BN) in order to optimize the diagnostic system. An efficient binary decision diagram with zero suppression (ZBDD) and diagnostic significance factor (DIF) is used. Automatic diagnosis based on the reliability analysis methodology (ADORA) using information about reliability during the design phase to build a diagnostic map is presented in [33,34].

An interesting issue concerning the use of induction trees is presented by Aljavarneh et al., where useful functions of engine defects were identified using the J48 decision tree algorithm [35]. The classification accuracy from the J48 algorithm, the best first tree algorithm, the random tree forest algorithm, the functional tree algorithm, and the linear tree algorithm are compared, and the best algorithm for a given system is recommended. Deptuła et al. [36] use a modular decision system based on graph networks parametrically playing an important role in the acoustic diagnostics of an internal combustion engine. Currently, the classification with the use of inductive decision trees is used in determining the most important faults. The algorithm, using decision trees, developed in this paper, analyzes the distribution of spectrum values around the analyzed harmonic for which a compatibility path is created for the given input (engine load). In this way, it is possible to identify the technical condition of the engine with a high probability, which is equivalent to the detection of a fault in the form of ignition or injection loss.

The applied vibro-acoustic methods in diagnosing the condition of a device use an inference method based on the knowledge of a domain expert. The authors of the study proposed to use the expert method in the form of decision trees. Such a solution makes it possible to extend the inference system with new attributes. Their increased number may positively influence the level of accuracy of the device state determination. The occurring differences in the amplitude of the fundamental harmonic and the variation of amplitude values for subsequent harmonics in the analyzed spectrum for a given failure are used to develop an expert base. The obtained expert database is used by the proposed system for conducting the inference analysis through multicriteria comparison of the analyzed data sample with the data contained in the database. Such a solution may allow for the construction of an extensive database, which can be used for diagnostic inference in the scope of diversified defects of the tested object using vibrations of its supporting structure as a source of diagnostic information.

## 2. Research Object

### 2.1. Measurement System

**Figure 1:**
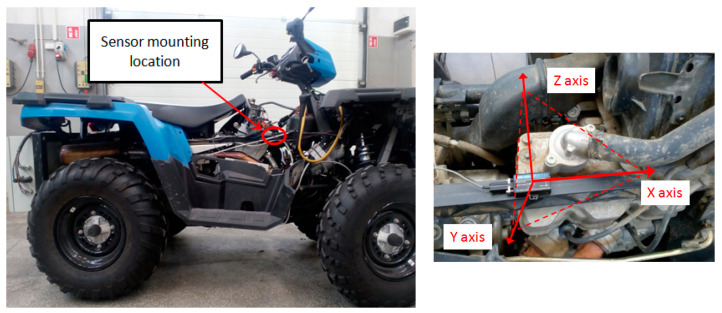
Sensor mounting location, sensor axis system.

The research involved the use of a Polaris off-road vehicle (the technical specifications of the test object are shown in Table 1) equipped with an original integrated measuring system consisting of a wireless data transmission system from the on-board CAN BUS network and an acceleration sensor (Figure 1). 

The wireless data transmission system uses diagnostic connector signals and allows data to be transferred from the vehicle to the recording computer using a radio transmitter (Ebyte Electronic Technology Co, Chengdu, China) operating in the 433 MHz band whose range is up to 1 km.

The sensor (3DM-GX3-25) (Lord Sensing, Villiston, ND, USA) was used to measure acceleration by a direct method. It is made in MEMS technology. Basic sensor data are presented in Table 2. The sensor is insensitive to the effects of internal noise of conditioning systems due to the use of a set of sensors with a pulse PWM output. It has a built-in processor which provides static and dynamic orientation of its measurement axes thanks to the algorithm of measurement synthesis.

**Figure 2:**
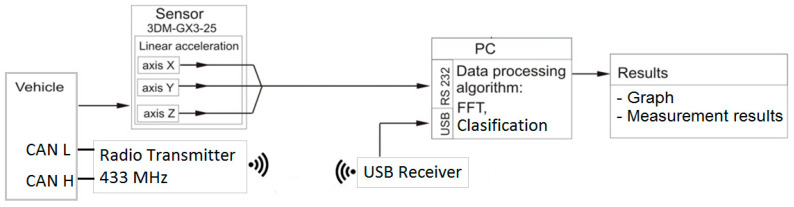
Placement of the 3DM-GX3-25 sensor on the vehicle during tests and block diagram of the measuring system.

The sensor for measuring vibrations has been mounted on a structural element (frame) of the tested vehicle. Such a solution is aimed at determining the possibility of using the vibrations of the supporting structure to identify the selected preset failures of the combustion engine. The diagnosis of the elastic-damping elements fixing the engine with the frame was carried out, confirming their correct operation. The directional setting of the axes of the sensor recording vibrations caused by the operating internal combustion engine relative to the engine crankshaft (x-longitudinal axis, y-transverse axis, and z-vertical axis) is shown in Figure 2.

### 2.2. Methodology

**Figure 3:**
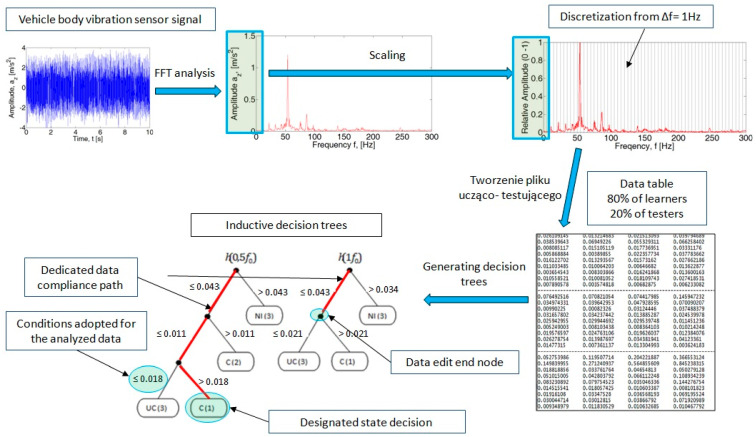
Example of acceleration sensor signal for a low-speed engine.

To identify periodic vibrations, selected signal analysis methods in the paper were used (Figure 3):

- analysis in the frequency domain FFT,
- scaling the spectrum to relative values,
- creating teaching and testing data files based on the scaled spectrum,
- inference methods based on decision trees.

To perform inference based on the decision tree, one needs the data recorded from the acceleration sensor and the on-board data transmission network of the actual machine, in this case all-terrain vehicle, which have been archived in a text file. In the next step, based on the FFT analysis of the recorded acceleration signal, an amplitude spectrum was determined and scaled to relative values in the range 0–1 and discretized. The discretization made it possible to determine the change in the distribution of the spectrum around the value of the analyzed harmonic defined on the basis of the engine’s rotational speed measurement. It normalizes the primary data, giving them the same weight and thus creating tables of the teaching and testing sets. Using the algorithm for generating inductive decision trees, it is possible to analyze the distribution of the spectrum around the analyzed harmonic. In this way, compatibility learning paths are created for the assumed technical condition of an internal combustion engine analyzed at a preset rotational engine speed. In the inference process, an analysis of a fragment of the recorded vibration signal of a combustion engine operating incorrectly at the same assumed rotational speed is performed. Comparative consistency of the pattern between the nodes of the decision tree allows one to determine the most probable technical condition of an internal combustion engine based on expert knowledge.

## 3. Measurement Signal and Analysis

**Figure 4:**
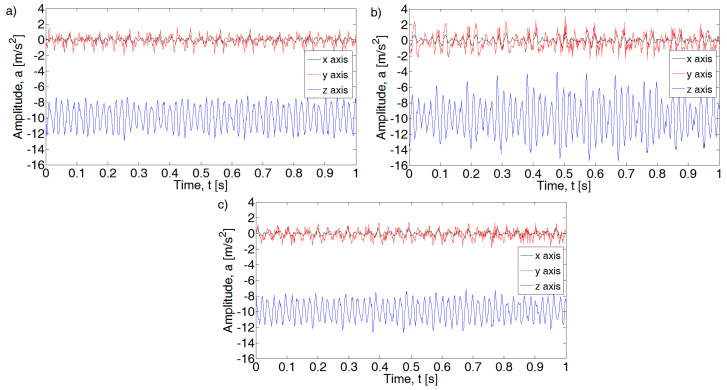
Example of acceleration sensor signal for 1250 rpm: (**a**) efficient engine, (**b**) pressure inefficient, and (**c**) injection inefficient.

**Figure 5:**
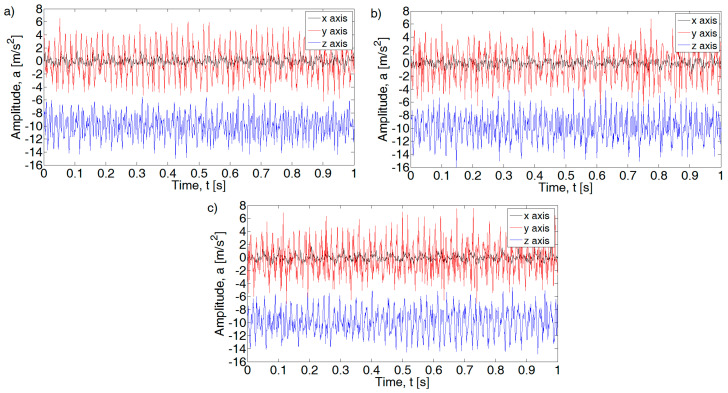
Example of acceleration sensor signal for 3500 rpm: (**a**) efficient engine, (**b**) pressure inefficient, and (**c**) injection inefficient.

For the analysis of the acceleration signal in the frequency domain, the waveforms at a constant speed of the crankshaft were used. In this way, for the speed of 1250 rpm, the spectrum of 30 cycles of the internal combustion engine’s operation (1 cycle = 2 shaft revolutions) was obtained. Fragments of histories of the recorded signals are shown in Figure 4 and Figure 5.

**Figure 6:**
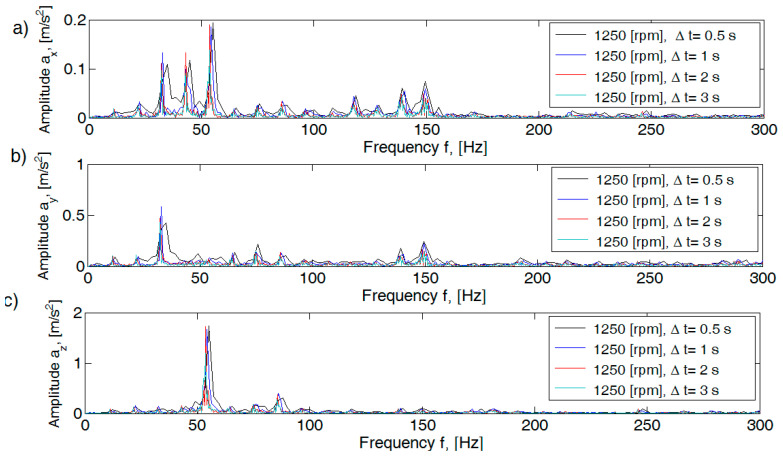
Amplitude spectra for assumed time intervals Δt: (**a**) along x axis; (**b**) along y axis; (**c**) along z axis.

For the recorded signal, the influence of the Hamming window on the Fourier transform shape was determined. Spectra obtained for assumed time intervals: Δt = 0.5; 1; 2; and 3 s are shown in Figure 6.

The obtained spectra for Δt = 1; 2; and 3 s show stable patterns for both the shape profile and peak values. In the author’s work [37], a detailed analysis of the selection of the time interval was made, where the time interval of Δt = 2 s for the measurement window established in 3 s was indicated for further analysis of the acceleration signal. 

**Figure 7:**
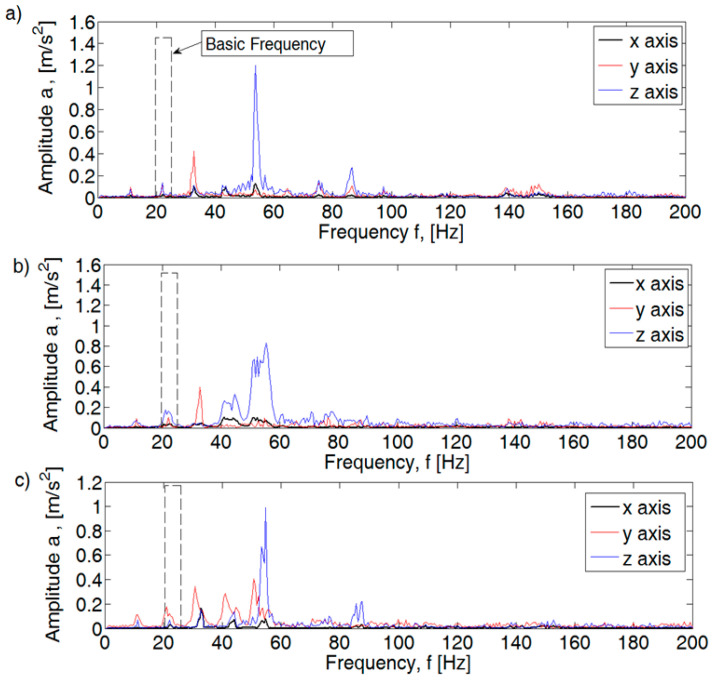
Fast Fourier Transformation FFT spectral amplitude for 1250 rpm combustion engine: (**a**) efficient, (**b**) injection inefficient, and (**c**) pressure inefficient.

For an efficient internal combustion engine at idle speed, the maximum acceleration values for the basic harmonic (f1) are ax = 0.0028 m/s^2^, ay = 0.19 m/s^2^, and az = 0.16 m/s^2^ (Figure 7). The highest acceleration values for the efficient motor were obtained for subsequent folds of the basic harmonic: y-axis (f 1.5) ay = 0.42 m/s^2^ and for the z-axis (f 2.5) az = 1.2 m/s^2^. Defects introduced in the form of a lack of injection into one cylinder or insufficient tightness of the spark plug caused a visible increase in the value of amplitudes for individual axes. The increase magnitude depends on the type of defects. In the case of an inefficient injection system, an increase is seen for the x axis for harmonics: f1, f2, and f2.5, for the y axis: f1, f2, f2.5, and f3, and the z axis: f2 and f2.5. The introduction of a defect in the form of a leak in the connection of the spark plug with the head did not introduce changes in the analyzed spectrum.

**Figure 8:**
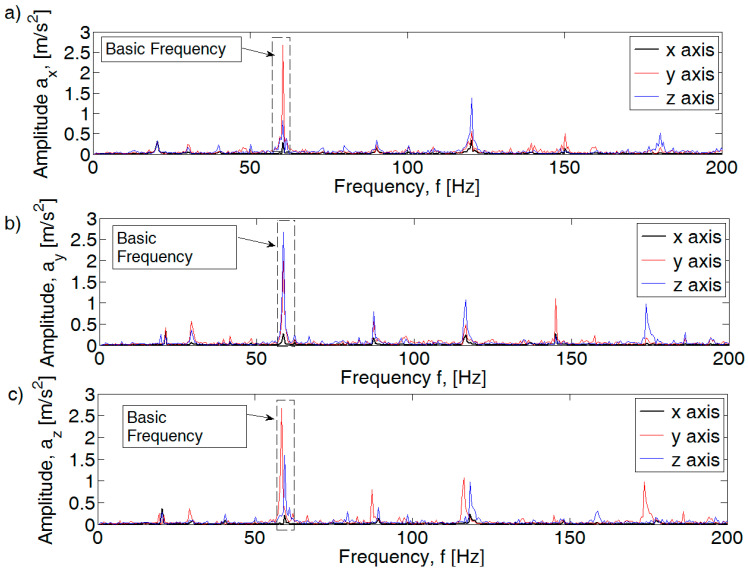
FFT spectral amplitude for 3500 rpm combustion engine: (**a**) efficient, (**b**) injection inefficient, and (**c**) pressure inefficient.

An increase in amplitude values is also visible for other engine speed ranges. An example of the waveform for the rotational speed of the crankshaft 3500 rpm is shown in Figure 8. Dominant amplitudes in the fundamental frequency range f_1_ as well as f_1.5_ and f_2_ are visible in the analyzed spectrum. For an efficient motor, the amplitude values for f_1_ are a_x_ = 0.28 m/s^2^, a_y_ = 2.75 m/s^2^, and a_z_ = 0.74 m/s^2^. An increase in the amplitudes of subsequent harmonics of the fundamental frequency (x axis and y axis) is also visible.

The introduced fault in the form of an inefficient injection system caused an increase in amplitude, which is visible for the harmonics f_1.5_ and f_2_ and f_1_ for the z axis. The leakage of the spark plug results in an increase in the amplitudes of the x and y axis accelerations for the f_2_ harmonic.

Based on the known rotational speed of the engine crankshaft, its basic frequency f1 was determined using a frequency range window of +/− 1.5 Hz. Then, dominant values of component acceleration amplitudes were determined for the *n*-order *n* = f_0.5_, f_1_, f_1.5_, f_2_, and f_2.5_. For the (x) axis, these values were determined as a_x0.5_, a_x1_, a_x1.5_, and a_xn_.

Then, relative values were determined for individual harmonics relative to the fundamental frequency. The obtained values take the designation Axn (*n*-order of the analyzed basic harmonic) for individual harmonic components of the signal spectrum (Figure 8). Based on Equations (3)–(5), the acceleration components for the measuring axis x, y, and z were determined for the selected rotational speed of the internal combustion engine.

### 3.1. Application of Induction Classification Trees in the Diagnosis of Internal Combustion Engine Damage

**Figure 9:**
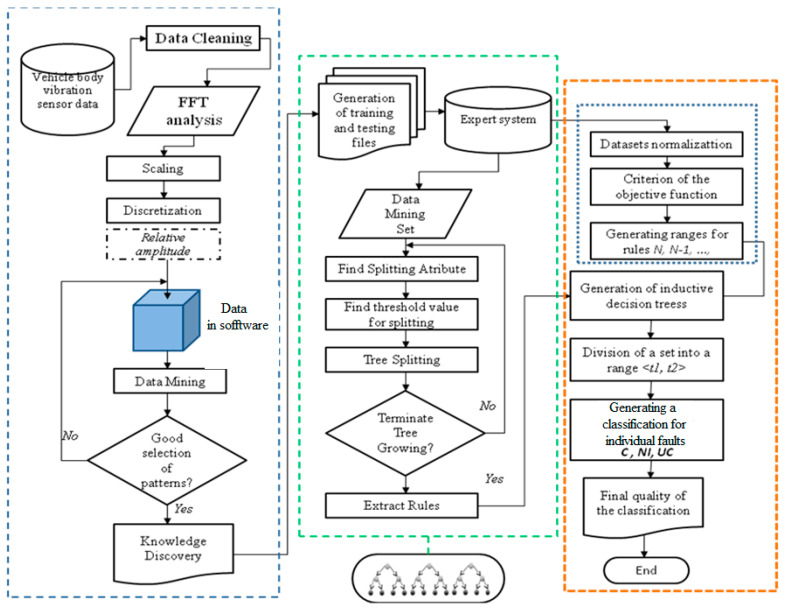
Classification system.

To analyze the data, an algorithm for generating inductive decision trees was used; the scheme of which is presented in Figure 9. The algorithm consists of three operating blocks:

Discovering knowledge—creating a database of registered signals from the acceleration sensor, FFT spectral analysis, and discretization;

Generating induction trees—the main algorithm for generating induction decision trees, including an expert database for teaching and testing

Minimizing the criterion function and a final search for the best solutions.

Most algorithms look for such rules by inductively generalizing the description of learning examples. The rules generated for each decision class should be satisfied by examples belonging to that class (so-called positive examples). At the same time, the rules should be met by none or only a few examples from other classes (the so-called negative examples) [25,26]. The main characteristics of machine learning are:

- the use of artificial intelligence methods and concepts (e.g., symbolic representation knowledge),
- the generalization (generalization) of the experience gained in the form of observation or decision examples,
- the explanation of the obtained generalizations and decisions made on their basis.

The task of the learning system is to obtain knowledge from a given subset of examples:

(1) L⊂U,

called the training set, which is most often a random sample from the population of all U objects. Objects are described by a set of features (attributes, variables):

(2) $$F=\left\{f_{1},f_{2},\ldots ,f_{m}\right\}$$

where *F*—family of functions, corresponding to the variables in the statistical interpretation.

The feature is a function that maps a set of objects (examples) to a set of values:

(3) $$f_{j}:U\to D^{-1}{\left(f_{j}\right)}^{2}$$

where $f_{j}(x_{i})$, f∈F-denotes the value of the *j*-*th* feature for the example x*_i_*

### 3.2. Generating Knowledge from Examples

As a result of training on a set of *L* examples described by *F* features, the machine-learning system generates the *h_F_ (L)* hypothesis. This hypothesis is a classifier. Knowledge representation languages can be divided into the following:

- symbolic (logical expressions, decision rules, decision trees, and semantic networks)
- subsymbolic (represented mainly by neural networks). Usually the knowledge representation language is imposed by the inductor. In a supervised environment, the hF (*L*) hypothesis generated by the inductor in the learning process is used to classify objects [9,10]. The hypothesis uniquely determines a certain partition PF (*U*). Then, one can determine the accuracy of classification for the set:
  (4) $$\eta (h,X)=\frac{1}{\left|X\right|}\left|X\cap \overset{n}{\underset{k=1}{\cup }}C_{k}\cap C_{k}^{’}\right|,C_{k}\in P(U),C_{k}^{’}\in P(U)$$
  where the error in classifying hypothesis h on the set of examples *X* can be determined as follows: ∈(h,X)=1−η(h,X).

Constructive induction is usually iterative, leading to successive changes in the representation space.

In the classification with the use of induction trees, it is assumed that the domain *U* (i.e., all our results), in which the attributes *u*_1_*, u*_2_*,..., u_n_*, (i.e., single measurements) are defined, is given a class of concepts *C* with a set of categories *C* and:

1. A leaf containing any category label with *z* ∈ *C* is a decision tree;
2. t: *X*→*i_t_* is a test carried out on the attribute values of examples with a set of possible results *i_t_=* *{ i*_1_*, i*_2_*, i*_3_*, ... i_m_}*; each is a single iteration (decision) in the tree search.

In the induction of decision trees, entropy determines the most significant attribute.

The criterion for choosing the attribute used to expand the tree is entropy as a measure of the information contained in the phenomenon, which can randomly take *n*-states:

(5) $$E=\sum_{i=0}^{n-1}\left(-p_{i}\log_{2}p_{i}\right),$$

where *p_i_*—is the probability of the appearance of the *i*th element of the set.

The information in the set of learning examples is equal to

(6) $$I(E)=-\sum_{i=1}^{\left|E\right|}\frac{\left|E_{i}\right|}{\left|E\right|}\cdot \log_{2}\left(\frac{\left|E_{i}\right|}{\left|E\right|}\right),$$

where

*E*—a collection of learning examples
$\left|E_{i}\right|$—a number of examples of the ith object,
|E|—a number of examples in the teaching set *E*.

The expected value of information after subdividing the set of examples *E* into subsets:

$E^{\left(m\right)},m=1,\ldots ,\left|V_{a}\right|$ in which the attribute a has the value of *V_m_*, specified as [12,13]:

where

*E*—a collection of learning examples
$\left|E^{\left(m\right)}\right|$—a number of examples after the *E* set is divided by the m value of the given attribute,
|E|—a number of examples in the training set *E.*

### 3.3. Identification of Defects Based on the Determined Spectral Characteristics of Engine Vibrations

Two-stage analysis was used: the set was divided into two parts *t*1 and *t2* (*t*1—the set of all examples belonging to 1250 rpm and *t2*—the set of all examples belonging to 3500 rpm) (Table 3).

Measurement data have been divided into classes:

(7) $$E=\frac{1}{2}\sum_{j=1}^{N}\sum_{i=1}^{n}{\left(x_{ij}-{\overset{⌢}{x}}_{ij}\right)}^{2},$$

**Figure 10:**
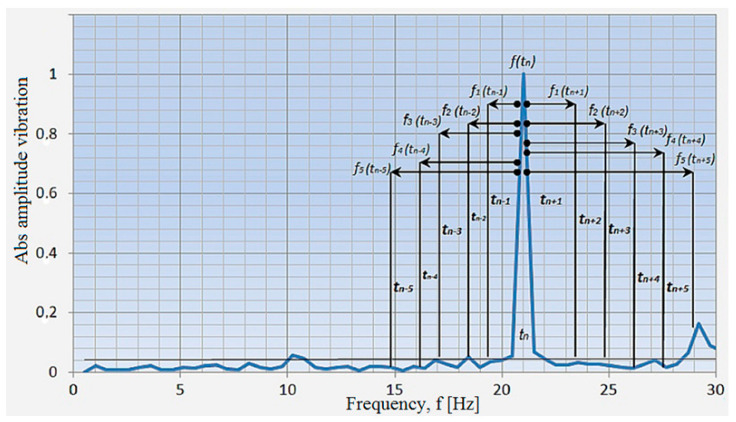
Interpretation of frame intervals for the analyzed amplitude spectrum.

Figure 10 shows an example of the division into classes in the classification of amplitude spectra for the assumed time intervals.

In the analysis including induction trees, five induction trees are obtained for the *t*1 range: (h*t*1 (0.5*f*_0_), h*t*1 (1*f*_0_), h*t*1 (1.5*f*_0_), h*t*1 (2*f*_0_), h*t*1 (2.5*f*_0_), and h*t*1 (3*f*_0_)) and five induction trees for the *t2* range: (h*t2* (0.5*f*_0_), h*t2* (1*f*_0_), h*t2* (1.5*f*_0_), h*t2* (2*f*_0_), h*t2* (2.5*f*_0_), and h*t2* (3*f*_0_).

**Figure 11:**
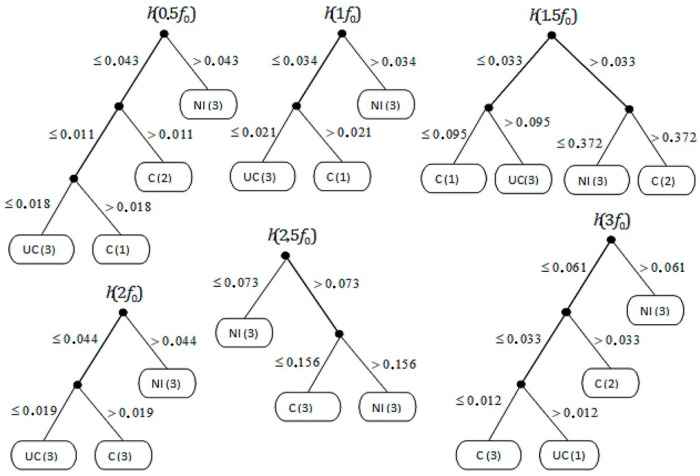
Inductive decision trees for *t*1, where C—efficient engine, NI—injection inefficient, and UC—pressure inefficient.

**Figure 12:**
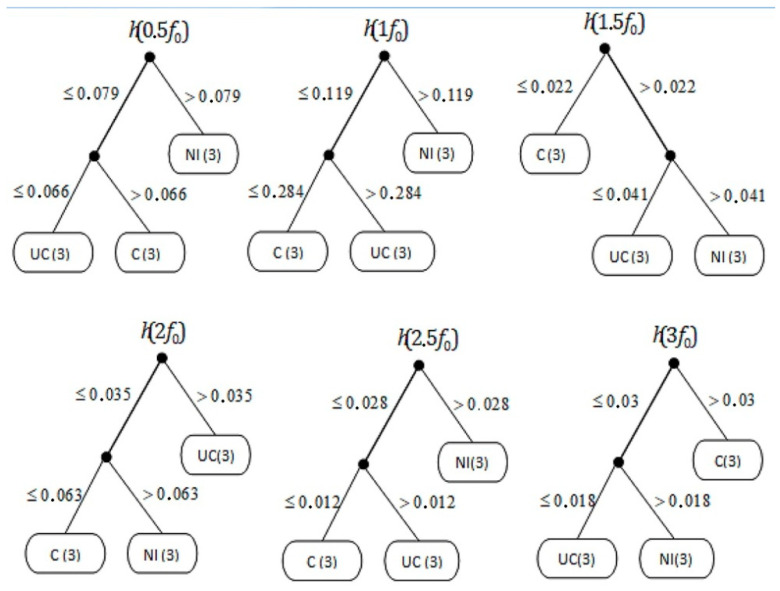
Inductive decision trees for *t2*, where C—efficient engine, NI—injection inefficient, and UC—pressure inefficient.

The decision system presented on Figure 11 and Figure 12 takes into account both conditions *t*1 and *t2* (). Due to this:

1. input attributes (in) are values for given frequencies *i*_1_*, i*_2_*, i*_3_*, i*_4_*, i*_5_*, i*_6_*, ..., i*_170_. In general, we have *n_k_* and for a given *n_k_*, where *k* = *1, 2, ..., N*.
2. *h*_1_*, h*_2_*, ... h_m_* are the hypotheses represented by DeTreex learning files leading to trees *T*_1_*, T*_2_*, ... T_m_*, respectively
3. the output attributes (out) represent the engine state, the test *t* with the result set *i_t_* = *{i*_1_*, i*_2_*, i*_3_*, ... i_m_}*.

The optimal response is assumed to be between 0–170 in the entire frequency range *F.*

The algorithm finds the optimal values of *i*_1_*, i*_2_*, i*_3_*, i*_4_*, i*_5_*, i*_6_*, …, i*_170_ and associates the engine state with the output:

(8) $$f_{N(t1)}=\prod_{n1}^{nk}\min\ldots \min\left\{n(h_{1},i_{1})+\ldots +n(h_{k},i_{k})\right\},$$

and

(9) $$f_{N(t2)}=\prod_{n1}^{nk}\min\ldots \min\left\{n(h_{1},i_{1})+\ldots +n(h_{k},i_{k})\right\},$$

where *t_1_*—is the determination of optimal values for 1250 rev/min (or the first stage of tree search) and *t_2_*—is the determination of the optimal values for 3500 rev/min (or the second stage of tree search). The change of state *i* to state *t_i+_*_1_ is described by the correlation:

(10) $$t_{i+1}=T(t_{i},h_{i}),$$

where transformation:

(11) $$T(t_{i},h_{i})=t_{i}-i_{i}h_{i},$$

The analysis of the *N*-stage process is carried out from the beginning to the end, searching all values from two sets of induction trees. The criterion function for the one-stage process *f*_1_ (*t*1) is only a function of the state *t_N_* and corresponds to the minimum *h* (*t_N_, i_N_*):

(12) $$f_{1}(t_{N})=\min h(t_{n},i_{i}),$$

For a two-step search, in which we want to find the optimal decision *i_N_-*_1_, we assume that the optimal value *f*_1_ *(t_N_)* and *f*_2_ *(t_N_)* for the *_N_*-*th* stage are known. The criterion function is now the sum of two components, while the optimal value of *i_N-_*_1_ is such that it corresponds to the minimum criterion function of a two-stage process:

(13) $$f_{2(t_{N-1})}=\prod_{i_{N-1}}^{N}\min\left\{n(t_{N-1},i_{N-1})+f_{1(t_{N})}\right\}=\min\left\{n(t_{N-1},i_{N-1})+f_{1(t_{N-1}-i_{N-1}h_{N-1})}\right\},$$

The procedure is analogous in the subsequent stages of tree searching: there is a recursive relationship of the *N*-stage decision-making process:

(14) $$f_{N-i+1(t_{i})}=\prod_{i_{N-1}}^{N}\min\left\{n(t_{i},i_{i})+f_{N-i}T[(t_{i},i_{i})\right\},$$

where *N*, *N*−1, ..., 1.

These dependencies allow building a search tree to generate a common database.

The use of the decision tree for backward inference, including the inductive classification system taking the form of the verification of a given hypothesis, runs from the hypothesis (goal) through the rules to the actual occurrence of the fault. In practice, it is done in such a way that the system tries to agree the hypothesis with a fact or a rule. In the case of disagreement, the algorithm searches for the next fact or rule and repeats the operation of finding whether the given value is in the area of diagnostics of the sought defect or its absence. Table 4 shows the probability of finding certain faults based on induction trees in terms of fault or its absence in the selected range of the internal combustion engine speed, i.e., *t*_1_ = 1250 rpm.

The analysis of the obtained results (Table 4) indicates which spectrum frequency range is most likely to identify engine malfunctions. For the analyzed speed of 1250 rpm, the highest probability of indicating the engine malfunction (Fault C) was obtained in the frequency range 1.5*f*_0_ at 44.9% (i.e., with 0.45 probability). A similar result is visible for frequencies 0.5*f*_0_ (44.47%) and 3*f*_0_ (44.47%). However, the probability of detecting the type of failure in these ranges is difficult to determine. For the frequency of 1.5 Hz, both signaled faults are within the same probability of detection of 27.55 (27%). The highest probability of detecting the fuel injection failure for one of the cylinders was obtained for the 2.5*f*_0_ range at 72.45%. The occurrence of compression pressure losses based on the presented method for 1250 rpm is very difficult. The highest probability of detecting a UC fault was obtained at the level of 27.55%, which proves the low effectiveness of UC fault identification.

For the analyzed speed of 3500 rpm, the highest probability of indicating the engine malfunction (Fault C) was obtained in the frequency range 1.5*f*_0_ at 44.9% and 3*f*_0_ at 44.9%. Similar to 1250 rpm, the probability of detecting the type of malfunction in these ranges is difficult to determine. The probability of indicating the occurrence of one of the faults is indicated by the algorithm at the level of 22.55% for each of the cases considered (Table 5). The highest probability of indicating the fuel injection failure for one of the cylinders was obtained for 0.5*f*_0_, 1*f*_0_ and 2.5 *f*_0_ at the level of 44.9%. The highest probability of indicating the abnormal pressure (UC) failure was obtained for 2*f*_0_ at 44.9%. 

## 4. Conclusions

On the basis of a multicriteria analysis using decision trees to identify the technical condition of the internal combustion engine for the occurring malfunctions in the combustion process, it is possible to detect an ignition and injection system fault. The analysis of the data table, using the created compatibility path of the tested engine block vibration signal, allowed for the following conclusions.

The proposed method of diagnosing selected engine faults based on advanced analysis of the engine block vibration signal allowed the determination of the technical condition using an intermediate signal.

The occurrence of faults in the ignition and injection system generates different paths of the engine block vibration signal analyzed in the developed decision tree algorithm.

The engine block vibration signal analyzed in X, Y, and Z domain has deterministic properties, so there are no simple methods of its analysis. Therefore, the applied methodology of engine block vibration signal analysis is divided into phases. In the first stage, FFT analysis was performed, and amplitude spectra were determined for subsequent dominant frequencies referring to the basic frequency as its harmonic components. These data constitute a learning and testing set for an efficient internal combustion engine. Then, using the algorithm for generating inductive decision trees, it is possible to analyze the distribution of harmonic frequency spectrum values. 

The use of expert knowledge for the developed algorithm of decision trees for the engine block vibration signal allows for the comparison between the nodes of the decision tree and for conclusions to be drawn on the technical condition of the examined object. In the analysis of the internal combustion engine operation under no-load conditions at a selected speed, it indicates that for each of the considered failures, there are dominant harmonics in the amplitude spectrum. The induction analysis allowed generating a forest of trees for 1250 speed and 3500 speed. The most important parameter selected during engine diagnostics is the fault “NI” pressure inefficient, occurring most often on the lowest floors of induction trees. Optimal analysis and classification should be made in the 3500 speed range. The classification process (generation) using trees is regular—the same height of all trees in the drawing at 3500 speed. Based on machine learning, it is possible to create a comprehensive diagnostic system. Comprehensive tree structures as the final result of the analysis of distribution allow for the presentation of direct operations of searching for the best variant in a completely formal way.

As a result of the analysis, the probability to detect an injection system malfunction amounts up to about 72% in the frequency spectrum for the next harmonic 2.5*f*_0._

## Figures and Tables

**Table 1:** Technical data of the test object (Polaris Sportsman XP 850).

Engine Type	Four-Stroke, Two-Cylinder, In-Line, Liquid-Cooled, SOHC
Engine displacement	850 cm^3^
Number of cylinders	2
Engine power	56.6 kW
Supporting structure	Welded steel frame made of closed profiles

**Table 2:** Acceleration sensor type 3DM-GX3-25 specifications.

Measurement Range	+/− 5 g
Nonlinearity	±0.1% fs
In-run bias stability	±0.04 mg
Initial bias error	±0.002 g
Scale factor stability	±0.05%
Noise density	80 μg/√Hz
Date output rate	866 Hz

**Table 3:** Adopted harmonic frequency ranges of the signal spectrum.

Rev/Min	Value f [Hz] with +/− Deviations			Rev/Min	Value f [Hz] with +/− Deviations			Cl.
1250	Lower Limit	Middle Limit	Upper Limit	3500	Lower Limit	Middle Limit	Upper Limit	
0.5*f*_0_	8.41666	10.4166	12.4166	0.5*f*_0_	27.1666	29.1666	31.1666	I
1*f*_0_	18.8333	20.8333	22.8333	1*f*_0_	56.3333	58.3333	60.3333	II
1.5*f*_0_	29.2500	31.2500	33.2500	1.5*f*_0_	85.5000	87.5000	89.5000	III
2*f*_0_	39.6666	41.6666	43.6666	2*f*_0_	114.6666	116.6666	118.8333	IV
2.5*f*_0_	50.0833	52.0833	54.0833	2.5*f*_0_	143.8333	145.8333	147.8333	V
3*f*_0_	60.6000	62.5000	64.5000	3*f*_0_	173.0000	175.0000	177.0000	VI

**Table 4:** Probability ranges for the diagnosis of the failure data for 1250 rpm, where C—efficient engine, NI—injection inefficient, UC—pressure inefficient, and VR—value range.

Rot. Speed [rpm]	Frequency Range	Fault C		Fault NI		Fault UC	
		VR	Prob. [%]	VR	Prob. [%]	VR	Prob. [%]
1250	0.5*f*_0_	<0.011–0.018>	44.47	>0.043	33.33	<0.018	22.23
	1*f*_0_	>0.021	27.55	>0.034	44.90	<0.021	27.55
	1.5*f*_0_	<0.095–0.373>	44.90	>0.372	27.55	>0.095	27.55
	2*f*_0_	>0.019	27.55	>0.044	44.90	<0.019	27.55
	2.5*f*_0_	<0.156	27.55	<0.073–0.156>	72.45	-	0
	3*f*_0_	<0.012–0.033>	44.47	>0.061	33.33	>0.012	22.23

**Table 5:** Probability ranges for the diagnosis of the defect data for 3500 rpm, where C—efficient engine, NI—injection inefficient, UC—pressure inefficient, and VR—value range.

Rot. Speed [rpm]	Frequency Range	Fault C		Fault NI		Fault UC	
		VR	Prob. [%]	VR	Prob. [%]	VR	Prob. [%]
3500	0.5*f*_0_	>0.066	27.55	>0.079	44.90	<0.066	22.55
	1*f*_0_	<0.284	27.55	>0.119	44.90	>0.284	22.55
	1.5*f*_0_	<0.022	44.90	>0.041	22.55	<0.041	22.55
	2*f*_0_	<0.063	22.55	>0.063	22.55	>0.035	44.90
	2.5*f*_0_	<0.012	22.55	>0.020	44.90	>0.012	22.55
	3*f*_0_	>0.030	44.90	>0.180	22.55	<0.018	22.55

## Data Availability

Not applicable.
